# Managing ocular surface disease: a common-sense approach

**Published:** 2017-02-10

**Authors:** Hon Shing Ong, John KG Dart

**Affiliations:** 1 Clinical and Research Fellow: Corneal and External Disease Service, Moorfields Eye Hospital, London, United Kingdom, **honshing@gmail.com**; 2 Professor and Consultant Ophthalmologist: Corneal and External Disease Service, Moorfields Eye Hospital, London, United Kingdom.

Many diseases can cause ocular surface disorders. The poster on pages 50–51 provides an overview of the most common diseases, and other articles in this issue focus on the management of individual diseases. In this article, the authors offer a systematic strategy for the **overall** management of ocular surface diseases.

When managing patients with an ocular surface condition, identifying the underlying disease is valuable (see pages 41–43 for guidance on assessment and diagnosis). However, diagnosis can sometimes be difficult or even impossible, as complex interactions exist between the different components of the ocular surface. A wide range of conditions can therefore result in similar functional effects at the ocular surface. These functional effects manifest as clinical signs common to several diseases, and include chronic punctate keratopathy, filamentary keratopathy, recurrent corneal erosion, bacterial conjunctivitis, culture-negative conjunctivitis, cicatrising (scarring) conjunctivitis, persistent epithelial defect, infectious keratitis, corneal melt and ocular surface failure (Figures 1A–G).

Fortunately, in the absence of a definite diagnosis, ocular surface diseases can usually still be managed effectively, provided the choice of approach and therapy is based on the functional effects observed and their severity. It is therefore important to have a systematic approach to the identification of functional effects and their severity (see Figure 2). Many of these functional effects are susceptible to a range of therapies, as discussed below.

**Note:** Ocular surface disorders often affect both eyes asymmetrically. Where patients present with unilateral disease, neoplasia – e.g. ocular surface squamous neoplasia (Figure 1H) –must be excluded.

## Management

### 1 Eliminate exacerbating factors

Eliminating exacerbating factors (if present) should be considered in all patients with ocular surface disease.

**Figure:**
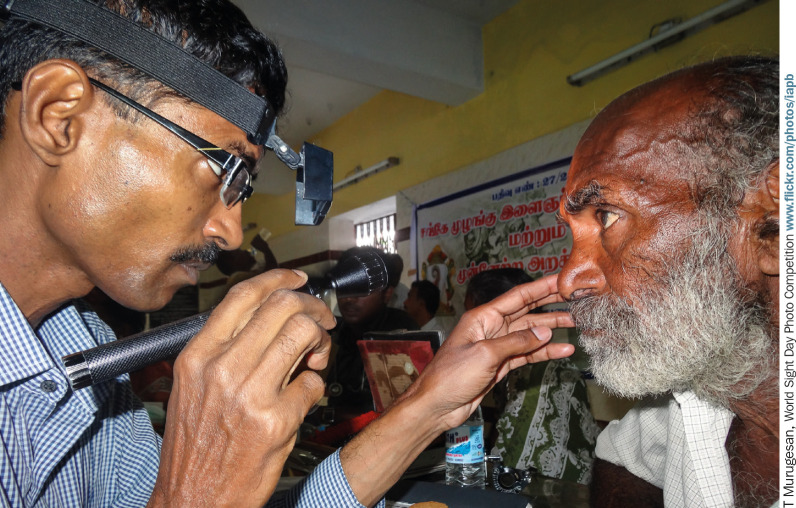
Involving patients is needed for good management of ocular surface diseases. INDIA

Ocular surface irritants have a negative effect on the recovery of the ocular surface.^1^ A common example is the use of glaucoma drops on a continuous basis. Unnecessary topical medications should be discontinued or systemic alternatives sought. If drops are needed, preservative-free formulations should be used where possible, especially if more than six drops are required daily. It may also be advisable to avoid using make-up and cosmetics on the eyelids and around the eye. Removal of exacerbating factors is particularly important in certain ocular surface diseases, such as allergic eye disease and Stevens-Johnson Syndrome.

**Blepharitis** is common and should be controlled to reduce its effects on tear film quality and the ocular surface.^2^ Lid hygiene (lid cleaning) removes crusts, debris and bacteria load on the lid margins in anterior blepharitis. Warm compresses and lid massage mechanically unblocks meibomian glands in posterior lid margin disease. One- to three-month courses of tetracycline class agents, such as doxycycline 100 mg once a day, are often helpful in controlling blepharitis in adults. ***Note:** doxycycline should **not** be given to children*. In children, or in adults where doxycycline is not tolerated, macrolides, such as erythromycin 250 mg twice a day, can be used. They are thought to improve meibomian gland dysfunction by altering their metabolism and secretion. Newer therapies, such as topical azithromycin 1.5% twice a day for 3 days, repeated weekly for 4–8 weeks, are also available.

Diseases of the eyelid and its adnexae (e.g. trichiasis, entropion) must be promptly addressed. Where appropriate, eyelid surgery should be considered.

### 2 Support ocular lubrication

An overlying physiological tear fluid is essential for a healthy ocular surface.^3^ Supporting the tear film should be considered in all cases of ocular surface disease, especially if the eye is dry. Lubricants not only serve as tear substitutes, they also help to dilute ocular surface irritants and reduce the shearing forces of the eyelids on the corneal epithelium. Many ocular lubricants are available. Some examples include hyaluronate, carmellose, hypromellose, polyvinyl alcohol, and paraffin. Lubricants with lipids or osmoprotectants (e.g. glycerine and L-Carnitine) are also available. Excess mucous can be treated with N-acetylcysteine drops.

Preservative-free lubricants are preferable for treating patients with ocular surface disease. Excessive use of drops with preservatives that are not diluted by normal tear flow can cause intolerance or ocular surface toxicity and impede ocular surface healing.

In aqueous-deficient dry eyes, punctal occlusion can prevent tear drainage and prolong the effects of tear substitutes. Punctal occlusion may exacerbate symptoms of blepharitis, so this must be treated beforehand. Permanent occlusion can be achieved by using punctal cautery. Parasympathomimetics such as oral pilocarpine can also be useful if tolerated. In more severe disease, autologous serum is beneficial, but this is expensive and not always readily available.

**Figure 1.**
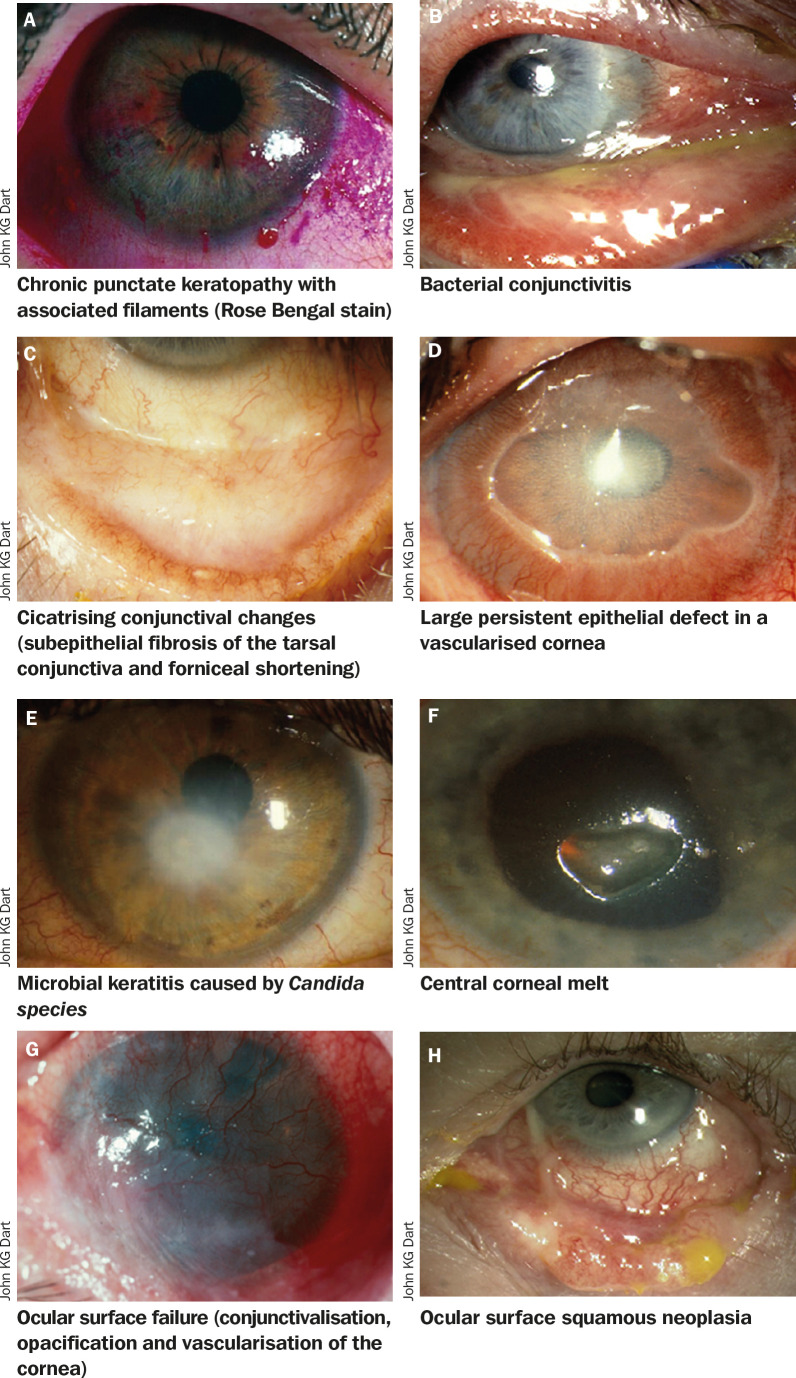
Functional effects (clinical signs) of ocular surface disorders

### 3 Consider therapeutic contact lenses

Therapeutic contact lenses (TCL) can be useful in severe dry eye diseases and persistent epithelial defects. Proposed mechanisms of action include modification of lid-tear-ocular surface interactions, retention of fibrin matrix on the surface of an injured cornea, and retention of tears under rigid lenses. In aqueous tear deficiency, hydrogel TCL should be avoided as the risk of infection is high. In very dry eyes, soft or silicone hydrogel TCL do not work well as they tighten up and reduce oxygen transmission. Rigid gas-permeable scierai TCL cover the cornea and most of the conjunctiva. This can prevent excessive tear evaporation and protects the ocular surface from abnormal lids.

**Figure 2.**
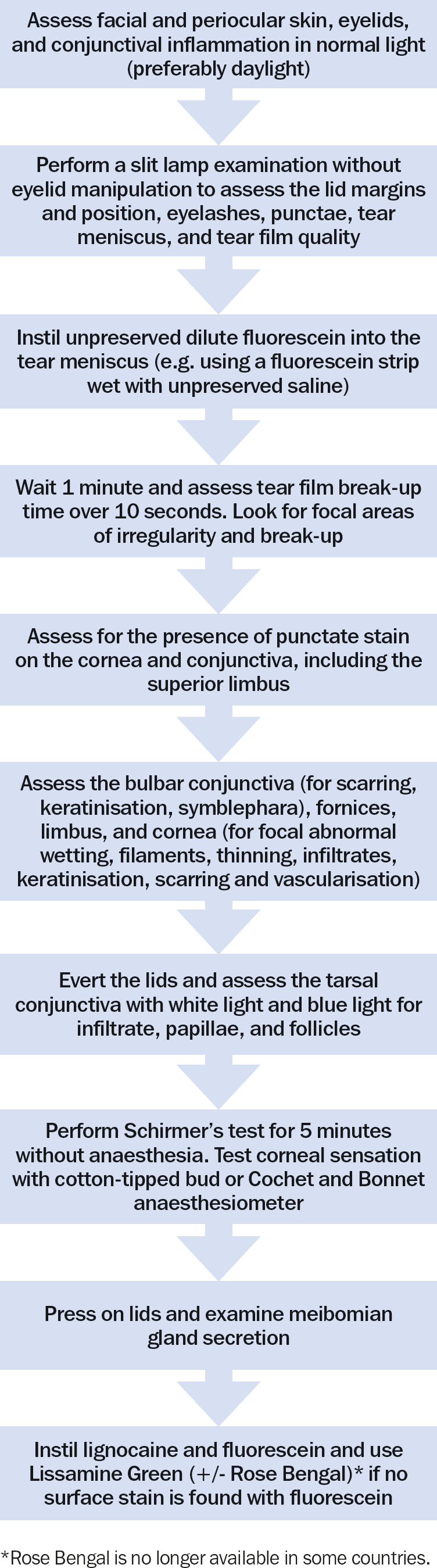
Ophthalmological assessment of a patient with ocular surface disease

### 4 Control ocular surface inflammation

**Figure 3.**
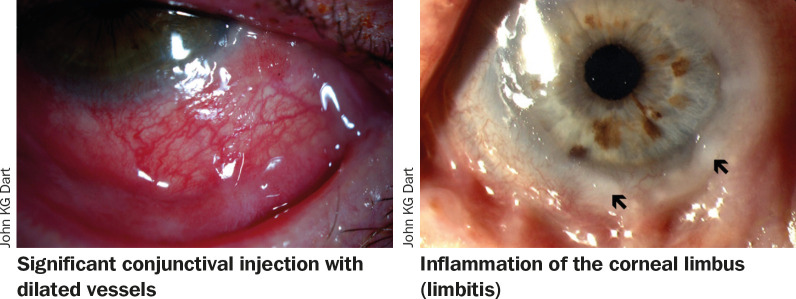
Severe ocular inflammation in ocular surface disease, namely cicatrising (scarring) conjunctivitis

An inflammatory component is seen in almost every form of ocular surface condition. Some clinical features of ocular surface inflammation include pain, conjunctival injection (redness), dilatation of conjunctival blood vessels, limbitis, conjunctival swelling (chemosis), redness and swelling of the eyelids (Figure 3).

Ocular surface inflammation is treatable. The choice of steroids depends on the severity of inflammation. In conditions where there is mild ocular surface inflammation, weak topical steroids (e.g. fluorometholone, or prednisolone 0.5% preservative free) can be used on an ‘as required’ basis or as short tapering courses. In severe inflammation (e.g. acute vernal keratoconjunctivitis), more potent topical steroids (e.g. dexamethasone 0.1%, or prednisolone 1%) are required. The frequency of drop administration is titrated according to disease severity. In cases where prolonged steroid use is anticipated, lenticular status, intraocular pressure, and assessment of the optic nerve head must be regularly documented to monitor for side effects such as cataract and glaucoma.

Topical ciclosporin A (various preparations) has been shown to be effective in several ocular surface disorders without the adverse effects of steroids. However, ciclosporin is often poorly tolerated during disease exacerbations and its full efficacy is only achieved several weeks from the initial dose. Ciclosporin has been shown to be better tolerated if introduced following a few weeks of treatment with topical steroids.^4^

Treatment of allergic eye disease (including acute, seasonal and perennial allergic conjunctivitis, vernal keratoconjunctivitis, and atopic keratoconjunctivitis) includes mast cell stabilisers (e.g. nedocromil, lodoxamide), antihistamines (e.g. emedastine, loratidine, chlorphenamine), or combined mast cell stabilisers/ antihistamine (e.g. olopatadine).

In severe ocular surface inflammation (e.g. corneal melts, mucous membrane pemphigoid), rapid immunosuppression is required to prevent visual loss.^5^ In these situations, immunosuppressive doses of steroids (e.g. prednisolone 1 mg/kg once a day and methylprednisolone 500–1,000 mg intravenous daily for 1–3 days) can be started and tapered off over 1–3 months once inflammation is controlled. Steroid-sparing drugs (e.g. mycophenolate, azathioprine, cyclophosphamide) should be started when a prolonged disease course is expected.

In ocular surface disease that is poorly controlled with topical therapy or where severe sub-acute inflammation persists, steroid-sparing therapy can be used without steroids. The use of such immunosuppressive agents requires specialist knowledge, monitoring, and facilities. These patients should be referred to specialist clinics if local medical services have insufficient support for the use of such agents.

### 5 Manage persistent corneal epithelial defects and microbial keratitis

Management of persistent corneal epithelial defects (PCED) is based on eliminating exacerbating factors, stimulating epithelialisation, improving epithelial stability, restoring the basement membrane, and renewing the epithelium. Nerve growth factor drops may be beneficial in cases of PCED secondary to neurotrophic keratopathy. Autologous serum and nerve growth factor treatments have both been shown to stimulate epithelialisation.

Microbial keratitis is a major complication in all patients with chronic ocular surface disorders. In any PCED, this must be excluded using appropriate microbiological techniques. Patients on topical steroids or systemic immunosuppressants may have an infection without a corneal infiltrate. Where infection is suspected, empirical treatment with a broad-spectrum antimicrobial should be initiated. Commonly, first-line treatment would include the use of fluoroquinolones (e.g. moxifloxacin 0.5%, levofloxacin 0.5%). Where fungal infection is suspected or diagnosed, steroid therapy must be discontinued and appropriate anti-fungal therapy commenced.

### 6 Surgical management

When non-surgical therapies fail to heal a PCED, lid closure with botulinum toxin injection or a temporary central tarsorrhaphy can be used to promote epithelial stability. In refractory PCED, improvement of the basement membrane can be achieved through human amniotic membrane grafts, lamellar keratectomy, or lamellar keratoplasty. Small perforations can be treated with cyanoacrylate glue and a contact lens. Therapeutic lamellar or penetrating keratoplasties are required for larger perforations.

Renewal of the epithelium through surface reconstruction can be considered if all of the above fail. Options for managing ocular surface failure due to limbal stem cell deficiency include allogenic or autologous limbal stem cell transplants.^6^

A conjunctival flap will sacrifice vision, but it reduces discomfort and ocular inflammation and promotes healing. If no conjunctiva is available due to scarring, a buccal mucous membrane graft can be used to provide a stable epithelium.

## Involve the patient

Successful management of ocular surface disorders can be difficult. Many conditions, such as allergic eye diseases, are chronic. Symptoms can often be controlled but not completely eliminated. Relapse and flare-ups are also common, and most treatments require the involvement of the patient over a long period of time.

It is important that patients are counselled before any treatment is started. They must understand the nature of their condition and the expected outcomes following treatment, as life-long therapies maybe needed. A management strategy should be agreed with patients and they must know how to access medical facilities in the event of a relapse.

## Conclusion

Many diseases can cause ocular surface disorders. Accurate diagnosis of the underlying condition may be difficult. In the absence of a definite diagnosis, identifying and treating the functional effects of the underlying disorder on the ocular surface is often sufficient.
